# Some New Uses for X-Rays

**Published:** 1920-05-01

**Authors:** 


### Some New Uses for X-rays.

In a lecture last week at the Royal Institution on the subject of "Recent Advances in X-ray Work," Major G. W. C. Kaye gave a highly interesting description of the gradual advance in this branch of physical science. To summarise an early history which is so well and so widely known is perhaps unnecessary, so a start may be made at the immediate pre-war period, when the invention having the most far-reaching effects was that of the Coolidge tube of 1913, which claims as its distinguishing characteristics a particularly high vacuum and a tungsten spiral as its cathode. The greatest change which its introduction has brought about lies in a remarkable reduction in the exposure time necessary to obtain a satisfactory *x*-ray photograph, and a number of excellent pictures were on view in which only one-hundredth of a second was needed. The good result of this power to obtain what practically amount to *x*-ray snapshots has already been felt in the medical world, as was recently exemplified in these pages in our review of an excellent volume on radiography of such difficult and constantly moving parts as the diaphragm and hepatic regions by Dr. Knox.

In technical industry, however, the advantages of ultra-rapid tube are even more manifest, and in this connection it was pointed out that an even more powerful *x*-ray unit had been invented and put in workable form on the Continental markets; but, as might perhaps be expected, the recent developments of these instruments tended to increase their fragility and therefore, in a certain degree, to limit their value in practice.

Of the lecturer's demonstration photographs, those showing fractures, embedded bullets, joint lesions, and the migrations of foreign bodies were perhaps more remarkable for their technical excellence than for any novelty in the use to which the rays had been applied.

A new and possibly valuable application to criminology was later outlined in connection with finger-print work. Briefly, the method consists of first cleaning the fatty substances from the skin surface by immersion in spirit, and then rubbing some lead compound well into all the epidermal irregularities. A radiograph then taken reveals with extraordinary accuracy the details of the prints, and we believe that the procedure is being favourably received by the Criminal Investigation Department as an improved substitute for the older direct methods.

A long and varied list of technical uses were dealt with in the lecture, and in a medical journal their chief claim to mention lies in the fact that the greatly improved details exhibited in the structure of articles photographed allows ready detection of flaws in all manner of large and small scientific machines, in which absolute accuracy is essential. Of more material developments illustrations galore were given, and the examples of testing the heating provisions in an airman's coat, the critical examination of steel-studded motor tyres, and the possibility of testing the golf balls used in the championship were calculated to draw the popular attention, if they did not immediately show to the audience a way in which the Briton of to-day can overcome, one and all, the uncertainties of this ultra-mechanical age.

